# *ACE* gene haplotypes and social networks: Using a biocultural framework to investigate blood pressure variation in African Americans

**DOI:** 10.1371/journal.pone.0204127

**Published:** 2018-09-18

**Authors:** Kia C. Fuller, Christopher McCarty, Cynthia Seaborn, Clarence C. Gravlee, Connie J. Mulligan

**Affiliations:** 1 Genetics and Genomics Graduate Program, University of Florida, Gainesville, Florida, United States of America; 2 University of Florida Genetics Institute, University of Florida, Gainesville, Florida, United States of America; 3 Department of Anthropology, University of Florida, Gainesville, Florida, United States of America; 4 Bureau of Economic and Business Research, University of Florida, Gainesville, Florida, United States of America; 5 Clinical Programs Center for Health Equity, Florida Agricultural & Mechanical University, Tallahassee, Florida, United States of America; 6 College of Pharmacy and Pharmaceutical Sciences, Florida Agricultural & Mechanical University, Tallahassee, Florida, United States of America; University of South Carolina, UNITED STATES

## Abstract

Deaths due to hypertension in the US are highest among African Americans, who have a higher prevalence of hypertension and more severe hypertensive symptoms. Research indicates that there are both genetic and sociocultural risk factors for hypertension. Racial disparities in hypertension also likely involve genetic and sociocultural factors, but the factors may interact and manifest differently across racial groups. Here we use a biocultural approach to integrate genetic and social network data to better understand variation in blood pressure. We assay genetic variation at the angiotensin I converting enzyme gene (*ACE*) and analyze social network composition and structure in African Americans living in Tallahassee, FL (n = 138). We demonstrate that models including both genetic and social network data explain significantly more variation in blood pressure and have better model diagnostics than do models including only one datatype. Specifically, optimal models for systolic and diastolic blood pressure explain a notable 35% and 21%, respectively, of blood pressure variation. Analysis of the social networks reveals that individuals whose networks are dominated by family connections and are more fragmented have higher blood pressure. Historically, family support has been associated with better mental and physical health, but our results suggest that those family connections can also take a toll on health. These findings raise compelling questions regarding the roles of genetics, family, and social environment in hypertension in the African American community and suggest that interactions among these factors may help explain racial disparities in hypertension more accurately than any of the factors alone.

## Introduction

Hypertension remains one of the most prevalent conditions in the US, affecting one in three adults [1]. Racial disparities in hypertension have been consistently reported with African Americans suffering from a higher prevalence of hypertension and higher rates of premature mortality than European Americans [1, 2]. As a complex disease, hypertension has both genetic and sociocultural risk factors. However, the basis of health disparities in hypertension is even less studied which makes identification of the determinants of hypertension and related racial disparities particularly complicated. Although the causes of these racial disparities in hypertension remain unclear, the support for a purely genetic contribution to cardiovascular disparities in African Americans vs European Americans is “essentially nil” [3]. Thus, studies that integrate both genetic and sociocultural data show promise for disentangling the complex effects of genetic variants and the sociocultural environment on hypertension and related health disparities. For example, Boulter *et al*. [4], identified a gene x environment interaction of the angiotensin I converting enzyme gene and vicarious discrimination and a threshold effect of vicarious racism that were only discovered because both genetic and sociocultural data were integrated in the study. In this study we focus on social network analysis as a reflection of the social environment in order to investigate which factors emerge as important when using a combined genetic and sociocultural approach.

Most of the evidence for the role of genetic and sociocultural factors in hypertension comes from studies that have focused on only one data type. Studies that focus on the genetic basis of hypertension have employed varied approaches, including genome wide association studies (GWAS) and candidate gene searches. Researchers have identified over 75 candidate genes for blood pressure regulation [5]. However, associations between variants in these candidate genes and hypertension do not always replicate across populations [6, 7]. Specifically, some of the variants have only been associated with hypertension in European American populations but not in non-European American populations [8, 9]. Furthermore, only a small fraction of studies have assayed these variants in non-European populations [10]. Here we investigate one of the strongest candidate genes, the angiotensin I converting enzyme gene, *ACE* [4]. *ACE* is the most studied blood pressure candidate gene and has been associated with hypertension in European and African American populations [4, 11]. The *ACE* gene is part of the renin-angiotensin system that regulates salt-water balance and blood pressure. ACE converts angiotensin I to angiotensin II, a hormone that helps regulate blood pressure and blood vessel constriction [12]. Inhibition of *ACE* gene activity stops the production of angiotensin II in the body and is used as a therapeutic to treat high blood pressure [13]. The *ACE Alu* insertion polymorphism is a 287 base-pair insertion in intron 16 that has been associated with increased risk of hypertension and myocardial infarction [14, 15].

The most widely studied sociocultural risk factor for hypertension is socioeconomic status (SES), which has been shown to generally predict systolic (SBP) and diastolic (DBP) blood pressure [16] in high income countries like the US. On average, individuals with lower SES have significantly higher rates of hypertension (both treated and untreated) than people with higher SES [17, 18]. Additionally, studies have shown that social norms and culture also play an important role in development of hypertension [19–21]. Specifically, while African Americans have a high prevalence of hypertension, they are less likely to engage in behaviors to prevent hypertension than other groups. This lack of preventative behavior is believed to be caused in large part by social and cultural norms within the African American community such as a tradition of food preparation that utilizes high levels of salt and a distrust of medical doctors that has been passed down for generations [21]. The importance of accounting for socio-cultural factors is also demonstrated by the fact that African Americans experience hypertension, with a prevalence of 37%, in greater numbers than Afro-Caribbeans, with a prevalence of 22% [22, 23]. If the main driving force behind racial disparities in hypertension was genetic, one would expect similar levels of hypertension in both populations as they share high proportions of West African ancestry. Thus, integrating genetic and sociocultural data in studies of hypertension in populations in the African Diaspora may lead to a more complete understanding of the disease etiology and racial disparity.

Social networks can be used to quantify aspects of the social environment because they are used to examine the ties, or relationships, in a person’s life [24]. Social networks have been used to study the transmission of infectious diseases, like HIV [25] and syphilis [26], as well as the spread of conditions not typically thought of as contagious like obesity, happiness, and gun violence [27–29]. Previous social network studies of blood pressure have identified higher SBP and DBP in association with lack of social support and having conflicted feelings, i.e. both negative and positive feelings, about people in the network [30, 31]. Additionally, larger network size and increased participation in club activities have been associated with lower SBP in a social network study of study of women and men [32]. The majority of studies do not speculate on the biological pathways through which these associations might manifest but Uchino et al. mention the stress response pathway as a viable mechanism [31].

Studies typically measure social networks by employing an elicitation prompt. An elicitation prompt asks participants to list people that they are tied to or have a relationship with; these individuals are referred to as alters. In an egocentric study such as the current study, participants are also asked if they believe their alters are tied to each other and, if so, how certain the participants are that these relationships between alters truly exist. Participants are often asked to provide information about alters, like their age, race, and perceived level of emotional closeness, and this information is used to develop measures of network characteristics like the level of racial diversity in a network or the compactness of a network.

Social networks may be particularly useful for studying racial health disparities because differences in network characteristics have been reported for different racial groups. On average in an urban setting, African Americans have smaller networks that include more family members than do European Americans [33]. In a study utilizing data from the National Health and Nutrition Examination Survey, a survey capturing data on African Americans, Hispanic Americans, and European Americans, social support was found to be a significant predictor of hypertension only in African Americans, and inclusion of social support in the model decreased racial differences in hypertension among all three populations [2].

Many studies examine a small number of alters (i.e. fewer than 10 people in a network) to focus on studying close ties (e.g. [34, 35]). In this study, we focus on a larger representation of an individual’s social environment and study both close and distant ties by requiring all participants to list 30 alters. Including 30 alters also allows us to examine both network composition and structure in relation to BP. Measures of network composition describe characteristics of individuals in a network and measures of network structure describe the ways in which individuals are connected, which are both important aspects of an individual’s social environment. Studies have shown that participants were able to easily list between 20 and 45 alters without undue burden [36, 37]. Furthermore, participants could easily evaluate all network ties, which is a much more involved task than just listing alters, if the alter pairs were queried in a systematic way, as they are in the present study [37].

The network measures used in this study are betweenness centrality and geodesic distance (both structural measures) and percent of alters who are family and percent of central positions occupied by family (both compositional measures). Betweenness centrality, or betweenness, is a measure of the number of shortest paths to each individual in the network. Geodesic distance, or distance, is a measure of the shortest path from one person to another [28]. Betweenness can be thought of as how often an alter connects one alter to another, while geodesic distance can be thought of as the most efficient (shortest) path between two alters. Percent of alters who are family and percent of central positions occupied by family are constructed measures focusing on the positioning and relative importance of family within a network.

In this study, we take a biocultural approach that integrates genetic data and social network characteristics to better understand blood pressure variation in 138 African Americans living in Tallahassee, Florida. Specifically, we test for the effect of genetic variants (42 single nucleotide polymorphisms [SNPs] and the *Alu* polymorphism at the *ACE* gene) and social network characteristics (measures of both network composition and structure based on 30 alters per participant, specifically betweenness, distance, percent of alters who are family members, and percent of central positions occupied by family) on blood pressure variation. The overarching goal is to investigate the factors among African Americans that may help explain racial disparities in complex diseases like hypertension.

## Methods

### Ethics statement

In accordance with the tenets of community-based participatory research (CBPR), we formed a steering committee (Health Equity Alliance of Tallahassee) to advise on all phases of our project, from its inception to participant recruitment and consenting to dissemination of results. The research protocol was approved by the University of Florida’s Institutional Review Boards (IRB-01 #364–2008 and IRB-02 #2007-U-469). Study participants were asked to read and sign written informed consent forms that included separate consents for participating in the interviews, saliva collection, finger-stick blood collection, requests for genetic ancestry reports as well as inclusion in future studies of other diseases. Signed written informed consent forms were collected from all participants and were stored in a locked filing cabinet in PI Gravlee’s office. Scanned copies of each participant’s signed written informed consent forms were also uploaded to a secure website, that only PIs Mulligan and Gravlee can access. Consent procedures were approved by the University of Florida’s Institutional Review Boards (IRB-01 #364–2008 and IRB-02 #2007-U-469).

### Sampling strategy and participant recruitment

As described in Boulter et al. [4], study participants came from a multistage probability sample of African American adults living in Tallahassee, FL. Census blocks were grouped using cluster analysis of neighborhood-level indicators of racial composition (percent of self-identified Black or African American residents) and material deprivation (e.g. percent of female-headed households, percent of vacant housing units, percent in poverty, median household income) (data from the U.S. Census Bureau’s American Community Survey). Within each cluster, block groups and then residential addresses were randomly selected. At first contact, we conducted a screening questionnaire to identify all eligible adults (self-identified African American, age 25–65) in each household. If more than one household member was eligible, we chose one at random, so as not to favor those more likely to open the door. We obtained informed consent from each participant before data collection began. The initial sample size was 185. Approximately 10% of participants (n = 20) did not give saliva samples and approximately 10% of participants (n = 19) did not participate in the second interview where social networks were collected. After accounting for incomplete sociocultural and genetic data, the final sample size was 138.

### Data and sample collection

Sociocultural data were collected via two face-to-face survey interviews with each participant. Interviews were conducted by members of the Tallahassee community who received training in interviewing techniques and collection of biological samples. The first interview lasted approximately 2–3 hours and collected a wealth of information on social stressors, financial status, neighborhood environment, food security, depressive symptoms, etc. and included age, sex, and use of antihypertensive medication. The second interview lasted about an hour, on average, and focused on eliciting data about people’s personal social networks (more detail below).

During the first interview, three blood pressure readings were taken using an oscillometric monitor (UA-787, A&D Medical, Tokyo, Japan). An average of the last two resting blood pressure readings was calculated for each individual and used in all analyses. Use of antihypertensive medication was corrected for by adding 10mmHg to the mean SBP and 5mmHg to the mean DBP readings of each participant, as previous researchers have suggested [38]. Thirty-one percent of individuals in our study indicated that they take medication to control their BP levels. To ensure the validity of this decision, we completed analyses using both the corrected BP measures and the uncorrected BP measures with blood pressure medication added as a variable. The results were the same regardless of which method of accounting for blood pressure medication was used. BMI was calculated using weight (measured by a digital scale) and height (measured with a stadiometer). Saliva samples for genetic analysis were also collected during the first interview.

### Genetic data and haplotype construction

Saliva samples were collected and stored using Oragene DNA Collection Kits (DNA Genotek®, Ontario, Canada). DNA was extracted per the manufacturer’s recommendations. DNA samples were genotyped for the *ACE Alu* polymorphism as described in Boulter et al. [4], and an additional 42 single nucleotide polymorphisms (SNPs) located in the *ACE* gene were assayed using a custom Affymetrix Axiom® Array(Affymetrix, Santa Clara, CA).

Haplotype construction was performed with Haploview [39] and PHASE [40] programs. Haploview was used to generate linkage disequilibrium (LD) blocks to determine which of the 42 SNPs and *Alu* polymorphism were inherited together as haplotypes. Ultimately, only two SNPs and the *Alu* polymorphism were found to be informative for regression analysis. PHASE was then used to identify the most likely genotype for each individual.

### Social network data

Ego-centered social network data were collected for each participant (Ego) using EgoNet [41]. Participants were prompted to “*name 30 people that you know by sight or by name that you could contact today if you needed to*.” All participants had to name 30 individuals, or alters. All information on alters was provided by the participants. Participants were asked to estimate the approximate age, sex, and skin tone of each alter. Information on the nature of their relationship with each alter was also queried with respect to the following categories: 1) blood family, 2) family by marriage, 3) spouse or significant other, 4) work, 5) school, 6) church 7) neighbor 8) hobby 9) organization, 10) through someone else or 11) other. Data on ties between alters of a participant’s network were collected using the prompt *“how likely is it that Alter A and Alter B talk to each other when you are not around*?*”* and participants were asked to answer this question using a rank system with 1 indicating “not at all likely” and 5 indicating “very likely”. Ties between alters were calculated using the EgoNet program. Ties were generated for pairs of alters based on the participant’s response to the aforementioned question. Alter pairs that were ranked a 4 “likely” or a 5 “very likely” by the participant were considered tied. Alter pairs that were ranked a 3 or lower did not have a tie generated. On average, participants answered with a 4 or 5 for 95.8% of alter pairs.

Betweenness centrality was calculated using the number of shortest paths between individuals in the network and mean betweenness centrality was calculated for each participant by averaging the betweenness centrality of each alter listed. Likewise, distance was measured by calculating the shortest path between one alter and another and mean distance was calculated by averaging each alter’s distance. Mean betweenness and mean distance are both continuous variables. Mean betweenness represents the extent to which a network is dominated by brokering nodes (in this study, the people listed by our participants). Mean distance can be used as an indication of the level of compactness of a network.

The percent of alters who are family was calculated by dividing the number of alters identified as blood family or family by marriage by the total number of alters (number of alters = 30). Spouses were classified as family. Percent of central positions occupied by family was calculated for each participant using three central positions in each network (the most between, the most close, and the most distant). The number of central positions occupied by alters identified as blood family or family by marriage was divided by the number of central positions (number of central positions = 3).

### Statistical analyses

#### SBP and DBP model building

The optimal models for SBP and DBP were generated through a series of multilinear regressions where age, BMI, and sex were included as standard covariates in all models. All non-normal data (specifically mean distance, mean betweenness, percent of central positions occupied by family members, and percent of alters who are family) were square root transformed prior to regression analysis. DBP was found to have a slightly non-linear relationship with one network measure that showed a significant association with DBP (percent of alters who are family). There were no significant interactions between percent of alters who are family and any of the standard covariates, genetic data or other network data and there was no threshold effect of percent of alters who are family on DBP, i.e. the effect of percent of alters who are family on DBP was not seen at a specific threshold, so we did not further transform measures of DBP or percent of alters who are family.

The first model, designated the base model, was generated using only the standard covariates. The second model, called the genetic model, included the standard covariates well plus genetic data (specifically, four *ACE* haplotypes). The third model, designated the social network model, included the standard covariates plus social network measures (specifically, mean betweenness, mean distance, percent of alters who are family members, and percent of central positions occupied by family). Alter characteristics (age, gender, emotional closeness) were tested in the social network model but did not improve model fit and therefore were not retained in the model. All statistical analyses were performed using the R statistical program [42].

#### Identification of optimal SBP and DBP models

AIC scores and adjusted R^2^ values were used to assess the fit of the model to the data; lower AIC and higher adjusted R^2^ values indicate a more optimal model. Using this method for each of the models, variables were added sequentially to the base model to determine if each variable improved the model, i.e. reduced the AIC and increased the adjusted R^2^value. If the variable was not found to improve the model, it was removed. Optimal models were chosen based on lowest AIC score and largest adjusted R^2^. The optimal models were then tested for statistically significant improvement relative to other models using ANOVA. All variables retained in optimal models for SBP and DBP are reported in the Statistical Analysis section of Results even though every variable was not significantly associated with BP within the model.

#### Additional statistical analyses

Tukey’s HSD test was used to evaluate if significant differences in SBP and DBP existed between *ACE* haplotypes. Bonferroni correction for multiple testing was used to assess the significance for individual variables and the models.

## Results

### Study demographics

Demographic characteristics for African Americans included in the study sample are detailed in Table 1. Mean age was 41 years old and women represented 66.7% of participants in the study. Mean BMI was 32.4, which is fairly high given that BMI measures of 25–30 are considered overweight and greater than 30 is considered obese [43]. In our study, 72.5% of participants had a BMI greater than 25, which is comparable to 76.3% of African Americans throughout the US who have BMIs greater than 25 [44]. After correction for the use of anti-hypertensive medication, 28% of participants had blood pressure readings in the hypertensive range (systolic blood pressure ≥ 140 mmHg) and 35% in the pre-hypertensive range (140 mmHg ≥ SBP ≥ 120 mmHg).

**Table 1:** Mean characteristics of study participants.

Characteristics	Male	Female	Total Sample
**N**	45	93	138
**Age, years (SD)**	41 (12.3)	41 (11.7)	41 (11.9)
**BMI (SD)**	28.8 (7.4)	34.2 (10.3)	32.4 (9.7)
**Systolic Blood Pressure, mmHg (SD)**	135.1 (22.4)	126.7 (20.1)	129.5 (21.2)
**Diastolic Blood Pressure, mmHg (SD)**	82.7 (13.7)	81.5 (13.3)	81.9 (13.4)

#### *ACE* haplotypes

**Fig 1:**
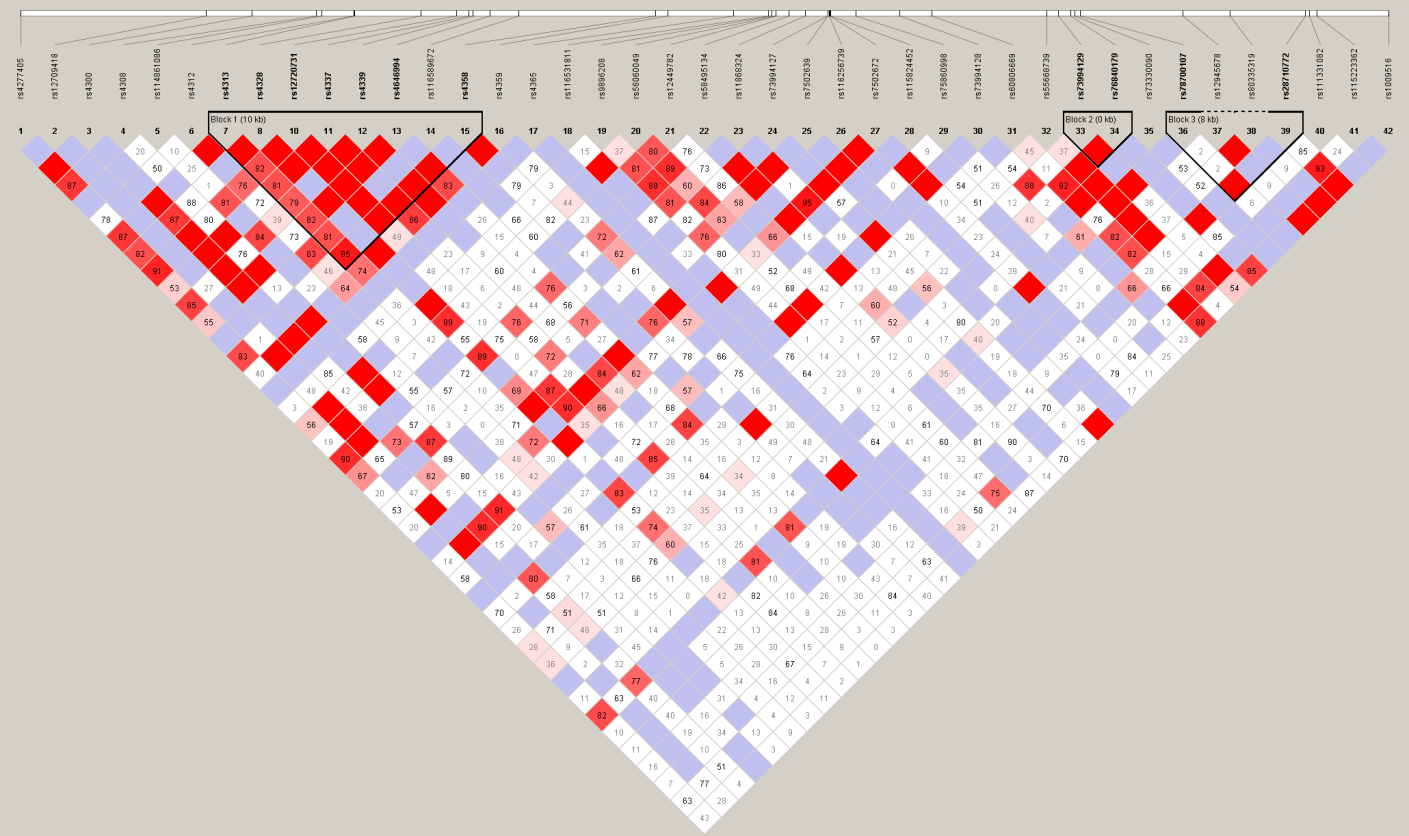
The LD block distribution of 31 SNPs and one *Alu* insertion polymorphism assayed in the *ACE* gene. Haplotype blocks are outlined in black. Darker shades of pink and red indicate a higher confidence in Hedrick’s multiallelic D’ measure of LD and a higher likelihood of LD. White and blue represent very low likelihoods of LD. Block 1 (far left) extends 10 kilobases (kb) and includes SNPs rs4313 to rs4358 and the *ACE Alu* insertion polymorphism (rs4646004).

Three linkage disequilibrium (LD) blocks were identified within the *ACE* gene (Fig 1). The majority of SNPs did not independently associate with BP and created haplotypes that were found at extremely low frequencies in the population. Additionally, many of the of the SNPs were non-polymorphic, with a minor allele frequency (MAF) < 5%, meaning there were only 1–2 haplotypes with these alleles. Ultimately, we focused on two SNPs (rs4313 and rs4337) and the *ACE Alu* insertion polymorphism, which occurred in block 1 and were in strong LD and adjacent to each other. Block 1 was the only LD block to significantly associate with BP and was the only LD block included in subsequent analyses. Four possible haplotypes based on block 1 markers were identified in the population (Table 2).

**Table 2:** *ACE* haplotypes.

Haplotype	rs4313—rs4337—*ACE Alu* polymorphisms*	Number of individuals
Haplotype 1	T-G-N	30
Haplotype 2	C-G-N	17
Haplotype 3	T-G-I	1
Haplotype 4	T-C-I	90

*The alleles in the haplotypes are ordered as follows: rs4313, rs4337, and *ACE Alu* polymorphism. For each haplotype, nucleobases cytosine, guanine and thymine are represented by ‘C’, ‘G’ and ‘T’, respectively, in the first and second positions of the haplotype while ‘I’ and ‘N’ represent the insertion and noninsertion alleles, respectively, of the *Alu* polymorphism in the third position.

### Social networks

**Fig 2:**
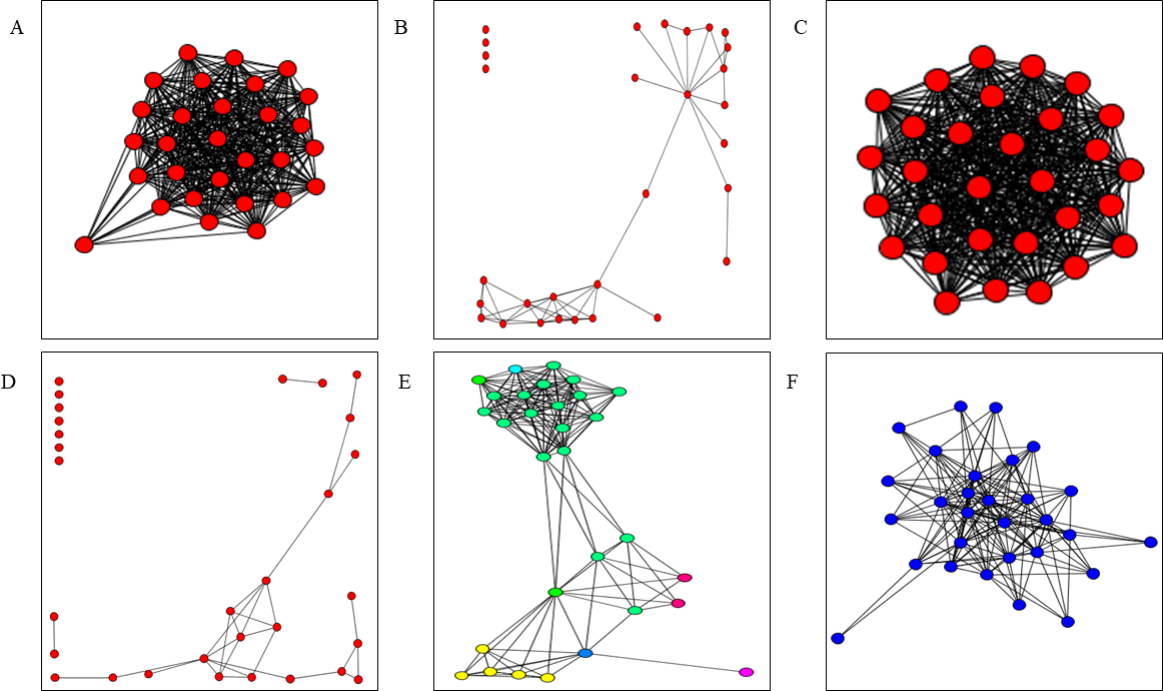
Examples of social networks from our study with representative measures. Unconnected red circles indicate isolated alters. **A)** Network with a low mean betweenness of 1.00. **B)** Network with a high mean betweenness of 23.27. **C)** Network with a low mean distance of 1.005. D) Network with a high mean distance of 3.214. **E)** Network with a low percentage of family members (shown in blue), 6.0%. **F)** Network with a high percentage of family members (shown in blue), 100%.

Social networks were analyzed for each of the 138 participants, who are referred to as ‘ego’. On average, approximately 50% of all alters listed were family members of the participants (Table 3). We examined measures of network structure (betweenness, distance, closeness), composition (percent of family members) and both structure and composition (location of family members in the network) individually to test for associations with BP. Individuals in our study had a mean betweenness of 6.9 and a mean distance of 1.76 (Table 3), indicating that most of our participants had cohesive, compact networks (see representative networks in Fig 2).

**Table 3:** Mean network characteristics of study participants across all networks.

Characteristics	Male	Female		Total
**Male alters: Female alters**	55:45	34:66	41:59	
**Mean alter age (SD)**	37 (9.5)	37.83 (10.2)	38.44 (9.9)	
**Mean % of alters who are family (SD)**	49.0% (28%)	51.0% (24%)	50.3% (25.5%)	
**Mean betweenness (SD)**	5.8 (5.58)	7.5 (5.85)	6.9 (5.80)	
**Mean distance (SD)**	1.66 (0.57)	1.80 (0.58)	1.76 (0.58)	

### Statistical analyses

In addition to age, sex, and BMI, the optimal model for SBP included the *ACE* haplotypes and two network characteristics: mean betweenness and percent of central positions occupied by family (Table 4). The optimal model explained 34.4% of variation in SBP (adjusted R^2^), compared to the base model (age, sex, BMI), which explained only 23.7% variation, and the AIC improved to 789 from 810. Furthermore, the optimal model with both genetic and network characteristics explained significantly more variance than did the optimal model with just genetic (p-value = 0.02) or network characteristics (p-value = 0.001) alone.

**Table 4:** SBP model selection using multilinear regression.

Base model				Genetic Model			Social Network Model			Optimal model		
Variable^^^	Co-efficient	Std. error	p-value^#^	Co-efficient	Std. error	p-value	Co-efficient	Std. error	p-value	Co-efficient	Std. error	p-value
Age	**0.55**	**0.13**	7.24*10^**−5**^	**0.55**	**0.13**	3.17*10^**−5**^	**0.54**	**0.13**	7.15*10^**−5**^	**0.55**	**0.13**	2.21*10^**−5**^
BMI	**0.75**	**0.17**	1.94*10^**−5**^	**0.72**	**0.16**	2.29*10^**−5**^	**0.76**	**0.17**	1.37*10^**−5**^	**0.74**	**0.16**	1.31*10^**−5**^
Sex (male = 1, female = 2)	**-12.92**	**3.47**	**0.0003**	**-12.16**	**3.34**	**0.0003**	**-14.70**	**3.45**	3.97*10^**−5**^	**-14.05**	**3.30**	4.06*10^**−5**^
Haplotype 2*				**-12.17**	**3.34**	**0.0004**				**-14.75**	**5.35**	**0.01**
Haplotype 3				-12.79	5.46	0.02				-32.42	17.78	0.07
Haplotype 4				**-13.24**	**3.75**	**0.0006**				**-13.70**	**3.64**	**0.0002**
Mean betweenness							2.43	1.27	0.056	2.59	1.22	0.04
Percent of central positions occupied by family							8.79	3.84	0.02	**9.39**	**3.66**	**0.01**
Adjusted R^2^	0.237			0.297			0.275			0.344		
AIC value	809.59			801.09			799.60			788.97		

^#^ P-values ≤ 0.01 indicate significance after Bonferroni correction for multiple testing (p = 0.05/ [1 haplotype block x 4 social network measures] = 0.01) and are shown in bold font.

* Haplotype 1 does not appear in the model because it is acting as a comparison group.

^^^ All variables that were tested in these models are listed in Methods.

In addition to the standard covariates, the optimal model for DBP included the *ACE* haplotypes and two network characteristics: percent of alters who are family and mean distance (Table 5). The optimal model explained 21.6% of DBP variation compared to the base model, which explained only 12.3% of variation, and the AIC improved to 683 from 702. Again, the optimal model with both genetic and network characteristics explained significantly more variance than did the optimal model with just genetic (p-value = 0.009) or network characteristics (p-value = 0.03) alone.

**Table 5:** DBP model selection using multilinear regression.

Base model				Genetic Model			Social Network Model			Optimal model		
Variable^^^	Co-efficient	Std. error	p-value^#^	Co-efficient	Std. error	p-value	Co-efficient	Std. error	p-value	Co-efficient	Std. error	p-value
Age	0.17	0.09	0.07	0.16	0.09	0.07	0.17	0.09	0.06	0.17	0.09	0.06
BMI	**0.48**	**0.12**	5.51*10^**−5**^	**0.44**	**0.11**	**0.0001**	**0.50**	**0.11**	2.16*10^**−5**^	**0.47**	**0.11**	5.34*10^**−5**^
Sex (male = 1, female = 2)	-3.95	2.35	0.10	-3.43	2.30	0.14	-5.16	2.33	0.03	-4.59	2.29	0.05
Haplotype 2*				-4.06	3.76	0.28				-5.52	3.70	0.14
Haplotype 3				-29.17	12.55	0.02				-25.34	12.36	0.04
Haplotype 4				-6.59	2.58	0.01				**-6.28**	**2.53**	**0.01**
Mean distance							10.80	5.12	0.04	9.60	5.07	0.06
Percent of alters who are family							**15.98**	**5.32**	**0.003**	**15.47**	**5.29**	**0.004**
Adjusted R^2^	0.123			0.1681			0.179			0.216		
AIC value	702.48			698.13			686.84			683.48		

^#^ P-values ≤ 0.01 indicate significance after Bonferroni correction for multiple testing (0.05/ [1 haplotype block x 4 social network measures] = 0.01) and are shown in bold font.

* Haplotype 1 does not appear in the model because it is acting as a comparison group.

^^^ All variables that were tested in these models are listed in methods.

Forty-two SNPs were tested but only two SNPs and the *ACE Alu* had sufficient variation to test for LD. Two *ACE* haplotypes, haplotypes 2 and 4, were significantly associated with SBP and haplotype 4 with DBP after Bonferroni correction (Tables 4 and 5). These results are somewhat consistent with Boulter et al (4), who found that the *ACE Alu* noninsertion allele was associated with higher SBP and DBP in this population. Standard ANOVA testing was used to further investigate the effect of *ACE* haplotypes on BP and revealed significant differences in mean SBP (p-value = 0.004) and DBP (p-value = 0.007) between haplotype groups. Tukey’s HSD testing showed that the most significant difference in SBP existed between haplotypes 1 and 4, which contain the noninsertion and insertion alleles, respectively (p-value = 0.01); haplotype 1 individuals had higher mean SBP than the rest of the sample population and significantly higher SBP than haplotype 4 individuals (difference of 13.42 mmHg, p-value = 0.002). Mean DBP was significantly higher for haplotype 1 individuals (difference of 35.76 mmHg, p-value = 0.02).

For SBP and DBP, two different network characteristics improved each model, although only one was significantly associated after Bonferroni correction, i.e. percent of central positions occupied by family was significantly associated with SBP (p-value = 0.01) and percent of alters who are family was significantly associated with DBP (p-value = 0.004). Specifically, a higher percentage of alters who are family members was significantly associated with higher DBP. A higher percentage of family members occupying two key network positions (highest level of closeness centrality and highest level of betweenness centrality) improved the SBP model and was associated with higher SBP. Conversely, participants who had diverse relationships with these position holders (i.e. a relationship reported as being different than family by marriage or by blood) had lower mean SBP. Overall, having many family members in the social network and in key positions in the network was associated with higher SBP and DBP.

## Discussion

The causes of hypertension and related health disparities have long been a source of study and debate among scientists and public health officials. The argument often pits genetic and sociocultural risk factors against one another, although it is known that both types of factors impact complex diseases like hypertension. Genetic and sociocultural factors also impact family history of disease, which has been investigated as a factor in health-related behaviors in African Americans [45]. In this study, we integrate genetic and sociocultural data to create a more comprehensive model of blood pressure variation in African Americans. We find that including both genetic and sociocultural data significantly improves both SBP and DBP models relative to models that include only one type of data. We hypothesize that genetic variants and network factors are important determinants of health, as possession of certain haplotypes and network makeup are associated with higher SBP and DBP, which are risk factors for diseases like cardiovascular disease.

The *ACE Alu* polymorphism is significantly associated with SBP, as reported in previous studies [4]. The *ACE* gene has been found to be essential in regulating blood pressure and salt levels [12]. We expand on these studies by assaying 42 SNPs in addition to the *Alu* polymorphism, and we increase the power of the study by creating *ACE* haplotypes that include all markers in linkage disequilibrium with each other (Fig 1). We find that haplotype 4 is significantly associated with a decrease in both SBP and DBP, and haplotype 2 is significantly associated with decreased SBP. Because haplotype 4 contained the *Alu* insertion allele these results are somewhat consistent with previous studies that also found an increase in blood pressure with the noninsertion allele [4]. However, haplotype 2 contained two copies of the noninsertion allele and was associated with decreased SBP which is not consistent with previous studies. The significance of this result may be artificially increased due to the relatively small number of individuals in the sample population with that haplotype.

Social networks are increasingly being used to investigate the effects of the social environment on health outcomes. Networks may reflect the cumulative effect of sociocultural factors that impact disease risk across a person’s life course. Christakis and Fowler [27] identified clustering of obese individuals in a network and found that the occurrence of a close alter becoming obese increased the likelihood that the study participant would become obese. Most network analyses have focused on network composition as opposed to network structure. Network composition refers to the characteristics of individuals who make up the network, e.g., age, sex, place of residence, or relationship with ego. Network structure refers to the organization of individuals in the network, e.g., mean betweenness, network centralization, or mean distance between alters. Mean betweenness provides a measure of how much a network is dominated by brokering alters; a low mean betweenness indicates that there is more cohesion across the network because there are multiple alters bridging gaps between groups (see Fig 2 for a representation of low mean betweenness). Central positions in the network are those that bridge gaps and are the positions through which alters must pass to reach other alters. Mean distance is a measure of how many paths an alter must take to reach another alter and reflects the density of a network; a low mean distance indicates a tight network where few paths are needed to reach everyone in the network (see Fig 2 for a representation of low mean distance). In our study, mean betweenness and mean distance had a positive association with blood pressure, indicating that a dense, cohesive network where information, support, or goods can be quickly passed to everyone is associated with lower blood pressure.

Our results on family members in the network are particularly striking. Previous studies suggest that social and familial support are positively associated with both mental and physical health outcomes [46]. Other studies have found that family relationships, especially marriage [47], are associated with several positive health outcomes. However, some studies have shown that family relationships can negatively impact one’s health [48]. Our study suggests that having many family members in a network is associated with higher blood pressure. Specifically, the percent of alters who are family members is significantly and positively correlated with DBP and an increased percent of central positions occupied by family members is significantly associated with increased SBP. Although the optimal models for SBP and DBP are not identical, both models suggest that having a high number of family members in a network is associated with higher blood pressure. Furthermore, the correlation between percent of alters who are family and percent of central positions occupied by family is 0.432 indicating a moderate positive relationship between the two measures, which is expected given that having more family members in a network increases the likelihood that family members occupy central positions. Our ability to detect these fine-grained associations reflects the fact that we looked at both network composition and structure (e.g. ‘percent of central positions occupied by family’ incorporates measures of both network composition and structure) and our study elicited more alters per participant than most studies (30 alters in our study vs ~5–10 in others) [34].

Our study may detect the underlying stress inherent in having a network comprised largely of family members. This possibility is consistent with several streams of research in the social sciences. First, social scientists have long observed that African Americans are more likely than others to live in extended-family households [49] and to be connected to other households through extended kin relations [50]. Second, for many African Americans, extended family members are crucial sources of psychological, emotional, and instrumental support in the context of economic and social marginalization [51, 52]. Although *access* to kin support may buffer social stressors and promote health, *providing* such support may itself be a stressor. For example, previous research shows that economic obligations to extended family members constrain the accumulation of wealth for middle-class African Americans and contribute to growing racial inequalities in household wealth [53]. Furthermore, the emotional labor of providing support to other family members may constitute a unique social stressor for African Americans, especially for women [54, 55].

### Limitations

While our study provided evidence for the usefulness of a using a biocultural framework to investigate complex phenotypes like blood pressure, our study was limited by its relatively small sample size and lack of information on diet and exercise. However, our results show that the sample size was sufficient to achieve significance under the more conservative Bonferroni correction. Furthermore, while studies have shown that diet and exercise affect blood pressure, they are time intensive data to collect and were not included in the already multi-hour interviews required of our participants. Our study also required participants to name 30 alters during the social network collection portion of the interviews. This may have increased respondent burden relative to listing fewer alters. However, information on 30 alters improved our structural measures, which were retained in the optimal models for SBP (mean betweenness) and DBP (mean distance). Finally, the sample collection area of this study was very focused, consisting of only African Americans in Tallahassee, Florida, which limited the variation that would be expected in diet and exercise.

### Conclusions

Our study provides support for a biocultural approach that integrates genetic and sociocultural data for a better understanding of complex diseases like hypertension. We demonstrate that models including both genetic and network data explain significantly more variation in blood pressure and have better model diagnostics than models that include only one type of data. In our study, the genetic and social network data are balanced in terms of complexity, unlike many studies, and this complexity allows us to make novel conclusions regarding the role of family as a factor that influences health. Specifically, having a large percentage of family members in the network and in central network positions is associated with higher blood pressure. Historically, family support has been associated with better mental and physical health, but our results suggest that those family connections can also take a toll on health. We anticipate that a biocultural approach will be particularly useful for identifying unique sociocultural causes of racial disparities in certain complex diseases.

## References

[1] Thorpe RJ Jr., Brandon DT, LaVeist TA. Social context as an explanation for race disparities in hypertension: findings from the Exploring Health Disparities in Integrated Communities (EHDIC) Study. Soc Sci Med. 2008;67(10):1604–11. 10.1016/j.socscimed.2008.07.002 18701200 PMC3521570

[2] Bell CN, Thorpe RJ, LaVeist TA. Race/Ethnicity and Hypertension: The Role of Social Support. Am J Hypertens. 2010;23(5):534–40. 10.1038/ajh.2010.28 20186126 PMC3102008

[3] Kaufman JS, Dolman L, Rushani D, Cooper RS. The Contribution of Genomic Research to Explaining Racial Disparities in Cardiovascular Disease: A Systematic Review. American Journal of Epidemiology. 2017;181(7):464–72. 10.1093/aje/kwu319 25731887

[4] Boulter AC, Florida Uo, Quinlan J, Florida Uo, Miró-Herrans AT, Austin UoT-, et al Interaction of Alu Polymorphisms and Novel Measures of Discrimination in Association with Blood Pressure in African Americans Living in Tallahassee. Human Biology. 2015;87(4). 10.13110/humanbiology.87.4.0295 27737583

[5] Sober S, Org E, Kepp K, Juhanson P, Eyheramendy S, Gieger C, et al Targeting 160 candidate genes for blood pressure regulation with a genome-wide genotyping array. PLoS One. 2009;4(6):e6034 10.1371/journal.pone.0006034 19562039 PMC2699027

[6] Adeyemo A, Gerry N, Chen G, Herbert A, Doumatey A, Huang H, et al A genome-wide association study of hypertension and blood pressure in African Americans. PLoS Genet. 2009;5(7):e1000564 10.1371/journal.pgen.1000564 19609347 PMC2702100

[7] Fox ER, and DoM, Young JH, Department of Medicine, and DoE, Li Y, et al Association of genetic variation with systolic and diastolic blood pressure among African Americans: the Candidate Gene Association Resource study. Human Molecular Genetics. 2011;20(11):2273–84. 10.1093/hmg/ddr092 21378095 PMC3090190

[8] Gupta S, Agrawal BK, Goel RK, Sehajpal PK. Angiotensin-converting enzyme gene polymorphism in hypertensive rural population of Haryana, India. J Emerg Trauma Shock. 2009;2(3):150–4. 10.4103/0974-2700.55323 20009302 PMC2776360

[9] Barley J, Blackwood A, Carter ND, Crews DE, Cruickshank JK, Jeffery S, et al Angiotensin converting enzyme insertion/deletion polymorphism: association with ethnic origin. J Hypertens. 1994;12(8):955–7. 7814855

[10] Franceschini N, Chasman DI, Cooper-DeHoff RM, Arnett DK. Genetics, Ancestry, and Hypertension: Implications for Targeted Antihypertensive Therapies. Curr Hypertens Rep. 2014;16(8):461 10.1007/s11906-014-0461-9 24903233 PMC4886553

[11] Duru K, Farrow S, Wang JM, Lockette W, Kurtz T. Frequency of a deletion polymorphism in the gene for angiotensin converting enzyme is increased in African-Americans with hypertension. Am J Hypertens. 1994;7(8):759–62. 7986468 10.1093/ajh/7.8.759

[12] Myerson S, Hemingway H, Budget R, Martin J, Humphries S, Montgomery H, et al Human angiotensin I-converting enzyme gene and endurance performance. Journal of Applied Physiology. 1999;87(4):1313–6. 10.1152/jappl.1999.87.4.1313 10517757

[13] Sweitzer NK. What Is an Angiotensin Converting Enzyme Inhibitor? Circulation. 2003;108(3). 10.1161/01.CIR.0000075957.16003.07 12876137

[14] Chung CM, Wang RY, Fann CS, Chen JW, Jong YS, Jou YS, et al Fine-mapping angiotensin-converting enzyme gene: separate QTLs identified for hypertension and for ACE activity. PLoS One. 2013;8(3):e56119 10.1371/journal.pone.0056119 23469169 PMC3587614

[15] Woo J, Tang NL, Leung J, Kwok T. The Alu polymorphism of angiotensin I converting enzyme (ACE) and atherosclerosis, incident chronic diseases and mortality in an elderly Chinese population. J Nutr Health Aging. 2012;16(3):262–8. 22456784 10.1007/s12603-011-0123-4 PMC12878106

[16] Kington RS, Smith JP. Socioeconomic status and racial and ethnic differences in functional status associated with chronic diseases. American Journal of Public Health. 1997;87(5). 10.2105/ajph.87.5.805 PMC1381054 9184510

[17] Bell AC, Adair LS, Popkin BM. Understanding the role of mediating risk factors and proxy effects in the association between socio-economic status and untreated hypertension. Soc Sci Med. 2004;59(2):275–83. 10.1016/j.socscimed.2003.10.028 15110419

[18] Leng B, Jin Y, Li G, Chen L, Jin N. Socioeconomic status and hypertension: a meta-analysis. J Hypertens. 2015;33(2):221–9. 10.1097/HJH.0000000000000428 25479029

[19] Fleury J, Lee SM. The Social Ecological Model and Physical Activity in African American Women. American Journal of Community Psychology. 2006;37(1):129. 16680541 10.1007/s10464-005-9002-7

[20] Dressler WW. Culture and the risk of disease. British Medical Bulletin. 2004;69(1):21–31. 15226194 10.1093/bmb/ldh020

[21] Peters RM, Aroian KJ, Flack JM. African American Culture and Hypertension Prevention. West J Nurs Res. 2006;28(7):831–63. 10.1177/0193945906289332 17056776 PMC2694441

[22] Lackland DT. Racial Differences in Hypertension: Implications for High Blood Pressure Management. Am J Med Sci. 2014;348(2):135–8. 10.1097/MAJ.0000000000000308 24983758 PMC4108512

[23] Bidulescu A, Francis DK, Ferguson TS, Bennett NR, Hennis AJM, Wilks R, et al Disparities in hypertension among black Caribbean populations: a scoping review by the U.S. Caribbean Alliance for Health Disparities Research Group (USCAHDR). International Journal for Equity in Health. 2015;14(1):125. 26541199 10.1186/s12939-015-0229-0 PMC4635613

[24] Borgatti SP, Everett MG, Johnson JC. Analyzing social networks: SAGE Publications Limited; 2013.

[25] Klovdahl AS, Potterat JJ, Woodhouse DE, Muth JB, Muth SQ, Darrow WW. Social networks and infectious disease: the Colorado Springs Study. Soc Sci Med. 1994;38(1):79–88. 8146718 10.1016/0277-9536(94)90302-6

[26] Fujimoto K, Flash CA, Kuhns LM, Kim JY, Schneider JA. Social networks as drivers of syphilis and HIV infection among young men who have sex with men. Sex Transm Infect. 2018. 10.1136/sextrans-2017-053288 PMC13002142 29440465

[27] Christakis NA, Fowler JH. The Spread of Obesity in a Large Social Network over 32 Years. The New England Journal of Medicine. 2009. 10.1056/NEJMsa066082 17652652

[28] Fowler JH, Christakis NA. Dynamic spread of happiness in a large social network: longitudinal analysis over 20 years in the Framingham Heart Study. The BMJ. 2008;337. 10.1136/bmj.a2338 PMC2600606 19056788

[29] Green B, Horel T, Papachristos AV. Modeling Contagion Through Social Networks to Explain and Predict Gunshot Violence in Chicago, 2006 to 2014. JAMA Internal Medicine. 2017;177(3):326–33. 10.1001/jamainternmed.2016.8245 28055070

[30] Holt-Lunstad J, Uchino BN, Smith TW, Olson-Cerny C, Nealey-Moore JB. Social relationships and ambulatory blood pressure: structural and qualitative predictors of cardiovascular function during everyday social interactions. Health Psychol. 2003;22(4):388–97. 12940395 10.1037/0278-6133.22.4.388

[31] Uchino BN, Cacioppo JT, Kiecolt-Glaser JK. The relationship between social support and physiological processes: A review with emphasis on underlying mechanisms and implications for health. Psychological Bulletin. 1996;119(3):488 8668748 10.1037/0033-2909.119.3.488

[32] Bland SH, Krogh V, Winkelstein W, Trevisan M. Social network and blood pressure: a population study. Psychosom Med. 1991;53(6):598–607. 1758945 10.1097/00006842-199111000-00002

[33] Ajrouch KJ, Antonucci TC, Janevic MR. Social networks among blacks and whites: the interaction between race and age. J Gerontol B Psychol Sci Soc Sci. 2001;56(2):S112–8. 11245365 10.1093/geronb/56.2.s112

[34] Cornwell EY, Waite LJ. Social Network Resources and Management of Hypertension. Journal of Health and Social Behavior. 2012;53(2):215–31. 10.1177/0022146512446832 22660826 PMC3727627

[35] Ajrouch KJ, Blandon AY, Antonucci TC. Social networks among men and women: the effects of age and socioeconomic status. J Gerontol B Psychol Sci Soc Sci. 2005;60(6):S311–s7. 16260713 10.1093/geronb/60.6.s311

[36] Golinelli D, Ryan G, Green HD, Kennedy DP, Tucker JS, Wenzel SL. Sampling to reduce respondent burden in personal network studies and its effect on estimates of structural measures. Field methods. 2010;22(3):217–30. 10.1177/1525822X10370796 21113275 PMC2990225

[37] McCarty C, Killworth PD, Rennell J. Impact of methods for reducing respondent burden on personal network structural measures. Social Networks. 2007;29(2):300–15.

[38] Tobin MD, Scurrah KJ, et al Adjusting for treatment effects in studies of quantitative traits: antihypertensive therapy and systolic blood pressure. Statistics in Medicine. 2005;24(19):2911–35. 10.1002/sim.2165 16152135

[39] Barrett JC, Fry B, Maller J, Daly MJ. Haploview: analysis and visualization of LD and haplotype maps. Bioinformatics. 2005;21(2):263–5. 10.1093/bioinformatics/bth457 15297300

[40] Stephens M, Smith NJ, Donnelly P. A New Statistical Method for Haplotype Reconstruction from Population Data—ScienceDirect. American Journal of Human Genetics. 2001;68(4):978–89. 10.1086/319501 11254454 PMC1275651

[41] McCarty C. Egonet: Personal Network Software. University of Florida 2003.

[42] Team RC. R: A Language and Environment for Statistical Computing. Vienna, Austria: R Foundation for Statistical Computing; 2016.

[43] Survey NHaNE. Healthy weight, overweight, and obesity among U.S. adults. National Health and Nutrition Examination Survey. 2003(03–0260).

[44] Statistics NCfH. Health United States 2016 Health United States 2016: With Chartbook on Trends in the Health of Americans. Hyattsville (MD): National Center for Health Statistics (US); 2016.

[45] Seaborn C, Suther S, Lee T, Kiros G-E, Becker A, Campbell E, et al Utilizing Genomics through Family Health History with the Theory of Planned Behavior: Prediction of Type 2 Diabetes Risk Factors and Preventive Behavior in an African American Population in Florida. Public Health Genomics. 2016;19(2):69–80. 10.1159/000443471 26845048

[46] Broadhead WE, Kaplan BH, James SA, Wagner EH, Schoenbach VJ, Grimson R, et al The epidemiologic evidence for a relationship between social support and health. American Journal of Epidemiology. 1983;117(5):521–37. 6342368 10.1093/oxfordjournals.aje.a113575

[47] Ross CE M John. Goldsteen Karen. The Impact of the Family on Health: The Decade in Review—download. Journal of Marriage and the Family,. 1990;52(4):1059–78.

[48] Haley WE, West CA, Wadley VG, Ford GR, White FA, Barrett JJ, et al Psychological, social, and health impact of caregiving: a comparison of black and white dementia family caregivers and noncaregivers. Psychol Aging. 1995;10(4):540–52. 8749581

[49] Taylor RJ, Chatters LM, Tucker BM, Lewis E. Developments in Research on Black Families: A Decade Review. Journal of Marriage and Family. 1990;52(4):993–1014.

[50] Dressler W, Alabama DoBSUo, Hoeppner SH, Alabama IfSSRUo, Pitts BJ, Alabama OoEDUo. Household Structure in a Southern Black Community. American Anthropologist. 1985;87(4):853–62.

[51] Stack CB. All Our Kin: Strategies For Survival In A Black Community: Basic Books; 1974.

[52] Billingsley A. Black families in white America. Englewood Cliffs, NJ: Prentice-Hall; 1968.

[53] Hardaway CR, McLoyd VC. Escaping Poverty and Securing Middle Class Status: How Race and Socioeconomic Status Shape Mobility Prospects for African Americans During the Transition to Adulthood. J Youth Adolesc. 2009;38(2):242–56. 10.1007/s10964-008-9354-z 19636721 PMC4108157

[54] Black AR, Peacock N. Pleasing the Masses: Messages for Daily Life Management in African American Women's Popular Media Sources. Am J Public Health. 2011;101(1):144–50. 10.2105/AJPH.2009.167817 21088274 PMC3000721

[55] Woods-Giscombe CL, Lobel M, Zimmer C, Wiley Cene C, Corbie-Smith G. Whose stress is making me sick? Network-stress and emotional distress in African-American women. Issues Ment Health Nurs. 2015;36(9):710–7. 10.3109/01612840.2015.1011759 26440874 PMC7220100

